# The LDL pathway regulates actomyosin ring dynamics necessary for optimal cell wound repair

**DOI:** 10.17912/micropub.biology.002019

**Published:** 2026-02-06

**Authors:** Geethika Burugupally, Mitsutoshi Nakamura, Cassandra Aarrestad, Susan M Parkhurst

**Affiliations:** 1 Basic Sciences Division, Fred Hutchinson Cancer Center, Seattle, WA, USA

## Abstract

Individual cells must rapidly repair any cortical damage from environmental or physiological stresses, to survive and to contribute to maintaining the proper function of tissues and organs. The formation of an actomyosin ring around the wound periphery is an important step in physically closing the cell wound. Here, we find that the Low-Density Lipoprotein (LDL) pathway, which is usually associated with plasma membrane homeostasis, is needed for optimal cell wound repair. In this context, the LDL pathway is required for robust actomyosin ring formation, revealing an unexpected role in regulating actin dynamics during cell wound repair.

**Figure 1. jeb and LpR2 knockdowns exhibit distinct phenotypes in cell wound repair:**
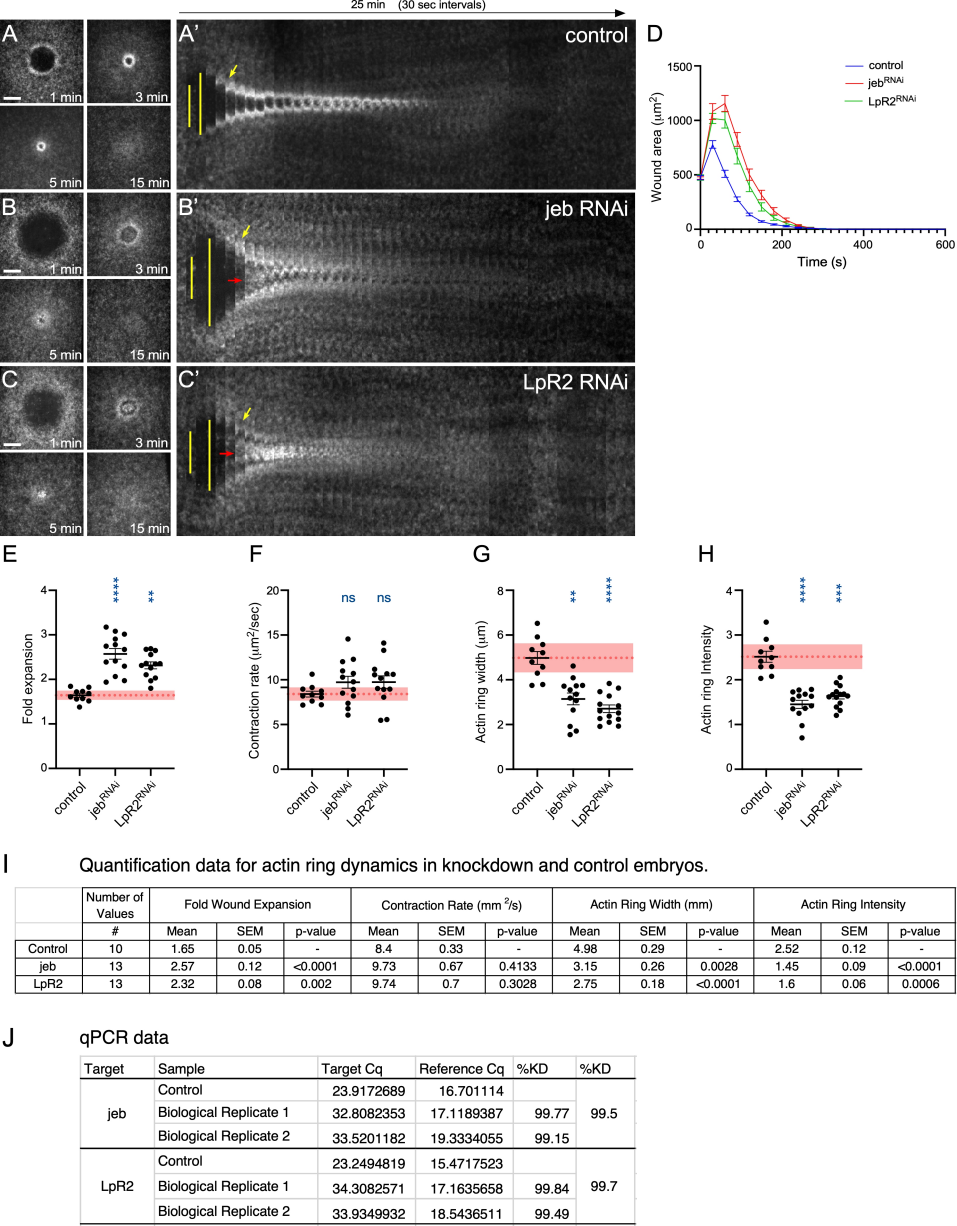
(A-I) Confocal max projection images of wounds generated in embryos expressing an actin marker (sGMCA) in: control (vermilion RNAi; A), jeb RNAi (B), and LpR2 RNAi (C). Scale bars: 20μm. (D) RNAi knockdown efficiency quantified using qPCR. (E-G) Quantification of the wound area over time for knockdowns shown in (A-C), respectively. (H-K) Quantification of actin ring dynamics in knockdowns. Quantification of fold wound expansion (H), wound contraction rate (I), actin ring width (J), and actin ring intensity (K). Black line and error bars represent mean ± SEM. Red dotted line and square represent mean ± 95% CI from control. Kruskal-Wallis test was performed: * p<0.05, ** p<0.01, *** p<0.001, **** p<0.0001, ns is not significant. (I) Quantification data for actin ring dynamics in knockdown and control embryos. (J) qPCR analyses of RNAi knockdown efficiency.

## Description

Single cell wound repair is a widely conserved process for restoring a cell after its cortex is damaged by physiological and environmental stresses to prevent cell death (Ammendolia et al., 2021; Dias & Nylandsted, 2021; Nakamura et al., 2018). Dynamic reorganization of the membrane and cortical cytoskeleton, followed by calcium influx at the wound site, regulates three major steps during cell wound repair: (1) vesicle recruitment to the wound site where they fuse with each other and the plasma membrane for rapid resealing of the wound hole; (2) cortical cytoskeleton reorganization around the wound to form an actomyosin ring for wound closure; and (3) membrane and cortical cytoskeleton remodeling after wound closure to return the cell to its unwounded state (Hui et al., 2022). Disease conditions like muscular dystrophy and diabetes disrupt membrane and cytoskeleton reorganization, or cause a fragile membrane that leads to frequent cell damage (Ammendolia et al., 2021; Dias & Nylandsted, 2021). Hence, understanding the mechanisms of cell wound repair in normal and disease conditions advances our knowledge for developing new therapies for related diseases and provides new insights into the fundamental regulation of the membrane and cytoskeleton during various cell behaviors.

Cholesterol is a major component of the plasma membrane, regulating its physical properties, such as bilayer fluidity and permeability, to maintain cellular homeostasis (Schade et al., 2020; Steck & Lange, 2018). Low-density lipoprotein (LDL) pathways play a role in controlling cholesterol levels in the membrane by circulating LDL particles that bind to LDL receptors on the cell surface and are internalized through receptor-mediated endocytosis. These LDL particles deliver cholesterol to endosomes and lysosomes, from which free cholesterol is released to membranes (Jeon & Blacklow, 2005; Litvinov et al., 2018; Luo et al., 2020). Previous studies have shown that disrupting LDL leads to a hypercholesterolemia, which slows repair of tissues (i.e., multicellular wound repair) and increases the risk of chronic, non-healing wounds by impairing inflammatory cells, angiogenesis, and fibroblast functions (Bogachkov et al., 2020; Gordts et al., 2014; Michalczyk et al., 2020; Potere et al., 2019). Lipid metabolism is an important metabolic perturbation in Duchenne Muscular Dystrophy (DMD) (Amor et al., 2021; White et al., 2020). Treating DMD tissue wounds with topical Simvastatin (lipid lowering drug) improved tissue wound repair parameters (Amor et al., 2021). While the LDL pathway is involved in tissue repair, we examined how the disruption of the LDL pathway affects single cell repair as it employs different components and mechanisms than multicellular/tissue repair (cf. Abreu-Blanco et al., 2012; Nakamura et al., 2018; Sonnemann & Bement, 2011).

To investigate the function of LDL pathways in single cell wound repair, we examined actomyosin ring dynamics throughout the repair process following laser wounding of jelly belly (jeb, LDL ligand) and lipophorin receptor 2 (LpR2, LDL receptor) knockdown embryos expressing a F-actin reporter (sGMCA). Using the GAL4-UAS system combined with the shRNA resource (Brand & Perrimon, 1993; Ni et al., 2011), jeb and LpR2 were efficiently knocked down in embryos (99.5% and 99.7% for jeb and LpR2, respectively) ( Figure 1J ). Only one RNAi line of each gene is available that efficiently knocked down expression in the germline. In control embryos, the wound size initially expands (fold wound expansion is 1.65±0.05), a robust actin ring forms at the wound edge, and then the wound closes ( Figure 1A- A', D, I). jeb and LpR2 knockdown embryos exhibit wound overexpansion (fold wound expansion is 2.57±0.12 and 2.32±0.08, respectively) ( Figure 1A- E, I). Neither jeb nor LpR2 affects the rate of wound closure compared to controls (9.73±0.67 and 9.74±0.7 for jeb and LpR2, respectively, compared to 8.4±0.33 for controls) ( Figure 1A- D, F, I). Both jeb and LpR2 knockdowns result in lower actin accumulation around the wound periphery with a smaller actin ring width (jeb: 3.15±0.26mm; LpR2: 2.75±0.18mm; control: 4.98±0.29mm), and lower actin ring intensity (jeb: 1.45±0.09; LpR2: 1.6±0.06; control: 2.52±0.12) (Figure1A-C’, G-I). Both jeb and LpR2 also exhibit F-actin accumulation inside the wound ( Figure 1B- C’). Hence, these results indicate that LDL pathways are required for proper actomyosin ring formation and optimal cell wound repair dynamics.

The impairment of tissue repair (i.e., multicellular wound repair) by disruption of the LDL pathway has been studied (Bogachkov et al., 2020; Gordts et al., 2014; Michalczyk et al., 2020; Potere et al., 2019). The focus in these studies has been on the role of the LDL pathway in membranes where it may function as a structural component of cell membranes needed for tissue integrity or in regulating signaling cascades that govern multicellular repair events such as inflammation and tissue remodeling. However, the components, machineries, and mechanisms required to close single cell wounds and multicellular wounds are different (Abreu-Blanco et al., 2012; Abreu-Blanco et al., 2011; Hui et al., 2022; Verboon & Parkhurst, 2015). We find that the LDL pathway is also required for efficient cell wound repair, but in this context is working to regulate proper actomyosin ring formation at the wound periphery. Rho family GTPases have been identified as key molecules in regulating the formation of the actomyosin ring during cell wound repair. Since previous studies showed that LDL and oxidized LDL levels affect Rho and Cdc42 (Oh et al., 2016; Singh et al., 2019; Zhang et al., 2017), the LDL pathway might regulate Rho family GTPases during cell wound repair needed to form a robust actomyosin ring. Alternatively, the LDL pathway might regulate actin dynamics through the Akt/PI3K pathway. We previously showed that knocking down insulin signaling components, including Akt and PI3K, disrupts a robust actomyosin ring formation and efficient cell wound repair (Nakamura et al., 2020). We found that insulin signaling is activated by cell wounds and regulates two actin regulators, Girdin and Profilin. The LDL pathway might be also activated upon cell wounding and regulate Girdin and Profilin through the Akt/PI3K pathway. Further study of the LDL and insulin pathways during cell wound repair will contribute to the development of new therapeutic approaches for chronic wounds and to improving repair efficiency under normal conditions.

## Methods

***Fly stocks and genetics***

Flies were cultured and crossed at 25ºC on yeast-cornmeal-molasses-malt medium. All fly stocks were obtained from the Bloomington *Drosophila* Stock Center (BDSC), treated with tetracycline, then tested by PCR to ensure that they did not harbor Wolbachia. To knockdown genes, RNAi lines were driven maternally using the GAL4-UAS system with P{matalpha4-GAL-VP16}V37 (BDSC #7063). The following RNAi lines were used: control: vermilion (v): y[1] v[1]; P{y[+t7.7] v[+t1.8]=TRiP.HMC03041}attP2 (BDSC #50641), &nbsp;jelly belly (jeb):&nbsp; y[1] sc[*] v[1] sev[21]; P{y[+t7.7] v[+t1.8]=TRiP.HMC04318}attP40 (BDSC #56022), and Lipophorin receptor 2 (LpR2): y[1] sc[*] v[1] sev[21]; P{y[+t7.7] v[+t1.8]=TRiP.HMS03722}attP2 (BDSC #54461). An actin reporter, sGMCA (spaghetti squash driven, moesin-alpha-helical-coiled and actin binding site fused to GFP), was used to follow wound repair dynamics of the cortical cytoskeleton (Kiehart et al., 2000). The crosses performed are:

**Table:** 

Generation	Female parent	Male parent
&nbsp;&nbsp;&nbsp;&nbsp; G0	sGMCA &nbsp;maternal Gal4	RNAi
&nbsp;&nbsp;&nbsp;&nbsp; G1	RNAi / +; sGMCA maternal Gal4 / +	RNAi / +; sGMCA maternal Gal4 / +

Embryos from the G1 cross were wounded or collected for qPCR analyses. Mutant analyses were performed at least twice from independent genetic crosses and ≥10 embryos were examined. Images representing the average phenotype were selected for figures.

***Embryo preparation and laser wounding***

Nuclear cycle (NC) 4-6 *Drosophila* embryos were collected from 0-30 min at room temperature (22ºC). Embryos were hand dechorionated, placed onto No. 1.5 coverslips coated with glue, and covered with Series 700 halocarbon oil (Halocarbon Products Corp). All wounds were generated with a pulsed nitrogen N2 Micropoint laser (Andor Technology Ltd., Concord, MA, USA) tuned to 435 nm and focused on the cortical surface of the embryo. A region of interest was selected in the lateral midsection of the embryo and ablation was controlled by MetaMorph. Ablation time was less than ~3s, and time-lapse imaging was initiated immediately.

***Microscopy***

Imaging was performed at room temperature (22ºC) using the following microscope: A Yokogawa CSU-X1 confocal spinning disk head mounted on a Nikon Eclipse Ti (Nikon Instruments, Melville NY,USA) with a 60x/1.4 NA objective lens and controlled by MetaMorph software. Images and videos were acquired with 488 nm and 561 nm, using an Andor iXon Ultra 888 EMCCD camera (Andor Technology Ltd., Concord, MA, USA). All images for cell wound repair were 17-20 µm stacks/0.25 µm steps. Images were acquired every 30 sec for 15 min and then every 60 sec for 25 min.

***Image processing, analysis, and quantification***

All images were analyzed with Fiji (Schindelin et al., 2012). Measurements of wound area, wound expansion rate, wound contraction rate, actin ring intensity and width were performed as previously described (Nakamura & Parkhurst, 2024; Stjepic et al., 2024).

***qPCR***

qPCR was performed as previously described (Nakamura & Parkhurst, 2024). Two biological replicates with two technical replicates each were performed. RpL32 was used as a reference gene. The % knockdown was calculated using the standard ∆∆Cq calculation method compared with control ( Figure 1J ).&nbsp; Primer sets used were:

RpL32: 5’-ATGCTAAGCTGTCGCACAAATG-3’ and 5’-GTTCGATCCGTAACCGATGT-3”

Jeb: 5’-GGAGCGAGAGCGACTAATGC-3’ and 5’-GACCGCGATGATTACTGCCC-3’

LpR2: 5’-GAAATAGCCTTGCATGTGATTGC-3’ and 5’-GTGGTAGACGGGATTCTCGAA-3’

&nbsp; ***Statistical analysis***

All statistical analysis was done using Prism 8 (GraphPad, San Diego, CA). Gene knockdowns were compared to the control, and statistical significance was calculated using a Kruskal Wallis test with *p* <0.05 considered significant.

## Reagents

**&nbsp;**

**Table:** 

**STRAIN**	**GENOTYPE**	**IDENTIFIER**
**sGMCA&nbsp;&nbsp; (GFP actin reporter)**	w[*]; P{w[+mC]=sGMCA}30	**Kiehart et al, 2000**
**matalpha4-GAL-VP16**	w[*]; P{w[+mC]=matalpha4-GAL-VP16}V37	**BDSC_7063**
**vermilion RNAi**	**y[1] v[1]; P{y[+t7.7] v[+t1.8]=TRiP.HMC03041}attP2**	**BDSC_50641**
**jeb RNAi**	y[1] sc[*] v[1] sev[21]; P{y[+t7.7] v[+t1.8]=TRiP.HMC04318}attP40	**BDSC_56022**
**LpR2 RNAi**	y[1] sc[*] v[1] sev[21]; P{y[+t7.7] v[+t1.8]=TRiP.HMS03722}attP2	**BDSC_54461**
**REAGENT**	**SOURCE**	**IDENTIFIER**
**iTaq Universal SYBR Green Supermix**	**Bio-Rad**	**Cat#1725120**
**iScript™ gDNA Clear cDNA Synthesis Kit**	**Bio-Rad**	**Cat#1725034**
**PRIMER NAME**	**SEQUENCE (5' -> 3')**	
**RpL32-F**	**ATGCTAAGCTGTCGCACAAATG**	
**RpL32-R**	**GTTCGATCCGTAACCGATGT**	
**Jeb-F**	**GGAGCGAGAGCGACTAATGC**	
**Jeb-R**	**GACCGCGATGATTACTGCCC**	
**LpR2-F**	**GAAATAGCCTTGCATGTGATTGC**	
**LpR2-R**	**GTGGTAGACGGGATTCTCGAA**	

**&nbsp;**
